# A case of intrasplenic displacement of an endoscopic double-pigtail stent as a treatment for laparoscopic sleeve gastrectomy leak

**DOI:** 10.1016/j.ijscr.2018.11.008

**Published:** 2018-11-13

**Authors:** Lucia Romano, Antonio Giuliani, Giovanni Cianca, Alessandra Di Sibio, Francesco Carlei, Gianfranco Amicucci, Mario Schietroma

**Affiliations:** a Department of Surgery, University of L’Aquila, L’Aquila, Italy; b Department of Radiology, University of L’Aquila, L’Aquila, Italy

**Keywords:** Case report, Gastric leak, Pigtail, Internal drainage, Intrasplenic migration

## Abstract

**Introduction:**

The most frequent and severe complication after laparoscopic sleeve gastrectomy is gastric leak. Nowadays, there is no specific standard recommendation for its management, but the endoscopic placement of a double-pigtail drain across the leak has been proven to be effective and minimally invasive. Stent displacement into the abdominal cavity is not common and intrasplenic migration is even more rare.

**Case presentation:**

We report a case of a 49-year-old woman with intrasplenic displacement of an endoscopic double-pigtail stent as a treatment for laparoscopic sleeve gastrectomy leak.

**Discussion:**

Pigtail drain migration involving the spleen is rare but may potentially expose the patient to the risk of parenchymal abscess or haemorrhage. Often the clinic does not indicate early this complication.

**Conclusion:**

We underline the need for a close radiologic follow-up, regardless of clinical conditions, in all patients treated with double-pigtail drain and its early removal in case of migration.

## Highlights

• The most frequent and severe complication after laparoscopic sleeve gastrectomy is gastric leak.
• The endoscopic placement of a double-pigtail drain across the leak has been proven to be effective and minimally invasive.
• Drain’s migration into the abdominal cavity is not common and its intrasplenic displacement is even more rare.
• Pigtail drain migration involving the spleen may expose the patient to the risk of parenchymal abscess or haemorrhage.

## 1 Introduction

The most frequent and severe complication after laparoscopic sleeve gastrectomy (LSG) is gastric leak (1–20%), that can lead to abdominal sepsis or chronic gastric fistula [1]. Nowadays, there is no specific standard recommendation for its management, but the current algorithm includes drainage, antibiotics and nutritional support [2]. Among the endoscopic procedures, the placement of an internal double-pigtail drain across the leak has been proven to be effective and minimally invasive, but not free of complications [3,4]. Drain’s migration into the abdominal cavity is not common and its intrasplenic displacement is even more rare. Only two cases are described in literature [3,5]. We report a new case because this complication could be, very likely, deadly. The work reported is in line with the SCARE criteria [6].

## 2 Case presentation

**Fig. 1:**
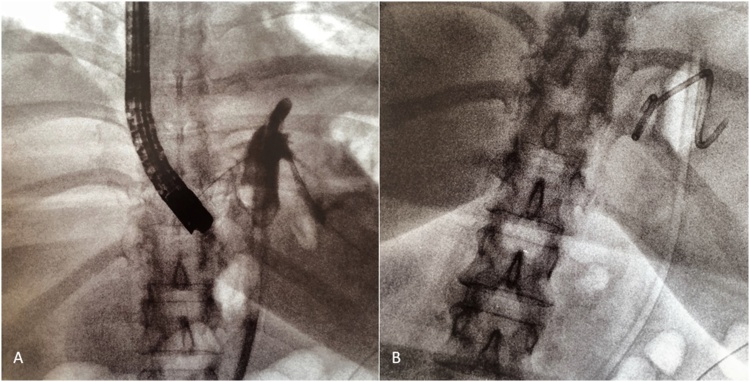
(A, B) – 8.5 Fr, 2-cm lenght double-pigtail stent was endoscopically delivered through the fistula orifice into the abdominal collection.

**Fig. 2:**
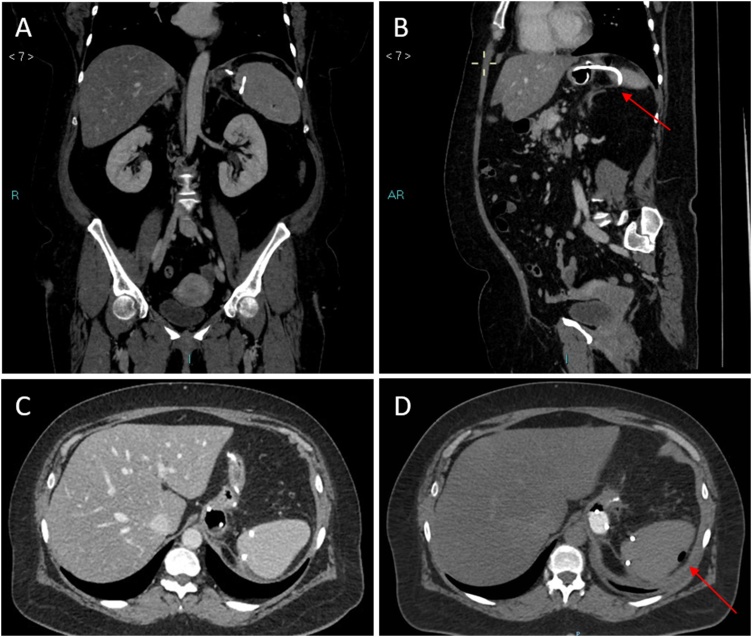
(A– D) – Abdominal computed tomographic scan with intravenous contrast, showing partial intrasplenic displacement of the double-pigtail stent, more evident in oblique projection along the axis of the vessel (red arrow in B). The presence of air in the subcapsular region confirmed intrasplenic displacement of the drain (red arrow in D).

The patient was a 49-year-old woman with a body mass index (BMI) of 40.8 kg/m^2^ who underwent a laparoscopic sleeve gastric resection on May 2018 in our centre. She did not take drugs, and had no history of significant diseases. Six days after surgery, she presented with tachycardia, dyspnea and fever (38 °C). The abdomen was tender, without signs of peritonitis. Blood tests revealed an increased white blood cell count of 11.84 migl/mmc, a C-reactive protein level of 10.23 mg/dl and a procalcitonin level of 0.74 ng/ml. A TC scan of the abdomen was performed after oral administration of water-soluble contrast medium, and it was suggestive of a proximal staple-line leak with abdominal collection in left hypochondrium and left lumbar. A surgical laparoscopic management was decided: two abdominal drain tubes were placed, and a 8.5 Fr, 2-cm lenght double-pigtail stent was endoscopically delivered by our reference endoscopists through the fistula orifice into the collection (Fig. 1). A repeat scan after administration of oral water-soluble contrast performed 10 days after endoscopic procedure revealed the presence of pigtail drainage with an *endo*-luminal end and an extra-luminal end, with an associated blind-ending cavity of about 3 cm. About 20 days after pigtail placement, a control CT scan was performed, which showed the external tip of the tube in close proximity to the medial side of the spleen, with partial intra-splenic displacement, but without parenchymal or vascular damage. The presence of air in the subcapsular region confirmed intrasplenic displacement of the drain (Fig. 2). At that time, the patient was completely asymptomatic and underwent endoscopic pigtail removal, without any bleeding. The last CT scan confirmed no laceration of the splenic parenchyma and no vessel injury or extravasation of contrast medium. At a 3-month follow-up the patient presented no further complications.

## 3 Discussion

In our paper we reported a case of partial intrasplenic displacement of a double-pigtail stent following internal drainage for a post-LSG leak, that required endoscopic removal. Also the case reported by Donatelli et al. [3], in which double-pigtail stent migration was responsible for the laceration of the splenic parenchyma without vessels injury, required endoscopic removal. In the case reported by Genser et al. [5], the migration of the drain was complicated by a splenic abscess and portal, splenic, and hepatic venous gas. In our case, as occured in the case described by Donatelli et al. [3], the patient had no symptoms and the discovery of the displaced drainage was occasional. Considering therefore the lack of reliability of the clinic and the severity of the damage that could be caused by drainage displacement, we would be led to strongly recommend the execution of a close radiologic follow-up.

## 4 Conclusion

Pigtail drain migration involving the spleen is rare but may potentially expose the patient to the risk of parenchymal abscess or haemorrhage. To early detect this condition, we suggests the need for a close radiologic follow-up, regardless of clinical conditions, in all patients treated with double-pigtail drain, and its early removal in case of migration.

## Conflicts of interest

No conflict of interest.

## Sources of funding

NO source of funding.

## Ethical approval

This study was approved by the Research Ethics Committee of the University of L’Aquila.

## Consent

The authors obtained patient consent to use all the images presented.

## Author contribution

Romano Lucia, Giuliani Antonio: Writing the paper.

Cianca Giovanni, Di Sibio Alessandra: Data collection and analysis.

Carlei Francesco, Amicucci Gianfranco, Schietroma Mario: Study concept.

Romano Lucia, Giuliani Antonio, Cianca Giovanni, Di Sibio Alessandra, Carlei Francesco, Amicucci Gianfranco, Schietroma Mario:Critical revision.

## Registration of research studies

NA.

## Guarantor

Prof. Mario Schietroma.

## Provenance and peer review

Not commissioned externally peer reviewed.
